# King Edward's Hospital Fund for London

**Published:** 1907-03-23

**Authors:** 


### KING EDWARD'S HOSPITAL FUND FOR LONDON.

### ANNUAL MEETING.

The annual meeting of the General Council of King Edward's Hospital Fund for London, to receive the Accounts and the Report of the General Council for the year 1906, was held at Marlborough House on March 15, the Prince of Wales (President of the Fund) in the chair.

Lord Rothschild (the Hon. Treasurer) presented the accounts.

The Prince of Wales moved the adoption of the Accounts, which was seconded by Sir Henry Burdett and supported by Sir Joseph Dimsdale, and carried unanimously.

Mr. Danvers Power read the Draft Report of the Council for the year 1906.

The Prince of Wales, in moving the adoption of the Report, said:—As at our last meeting in December I went fully into the various questions affecting the Fund, I do not propose to do more to-day than to formally move the adoption of the Report. I consider the meeting of the Council to pass the distribution of our funds to be the most important one of the year, and I therefore intend for the future to keep any remarks I may have to make for that occasion. But I should like to take this opportunity of thanking Sir John Craggs, who was one of our Honorary Secretaries from the foundation of the Fund until last June, for his invaluable services which have been of the greatest assistance to the Fund, and have involved the expenditure of an enormous amount of time and trouble on his part. We cannot be too grateful to him for what he has done for us, and we are all, I am sure, very glad to know that we shall still have the benefit of his advice as a member of the Council and Committees on which he serves. I now move the adoption of the Report.

The resolution was seconded by Sir William Collins, supported by Mr. Henry Morris (President of the Royal College of Surgeons) and Sir John Craggs, and carried unanimously.

The progress of the Bill for incorporating the Fund was reported.

The Lord Mayor (Sir W. P. Treloar) moved, and the Chairman of the County Council (Mr. H. Percy Harris) seconded, a vote of thanks to the Prince of Wales for presiding, which His Royal Highness briefly acknowledged.

The proceedings then terminated.

